# Draft genome sequence of *Bacillus azotoformans* MEV2011, a (Co-) denitrifying strain unable to grow with oxygen

**DOI:** 10.1186/1944-3277-9-23

**Published:** 2015-01-21

**Authors:** Maja Nielsen, Lars Schreiber, Kai Finster, Andreas Schramm

**Affiliations:** 1 Section for Microbiology, Department of Bioscience, Aarhus University, Aarhus, Denmark; 2 Center for Geomicrobiology, Department of Bioscience, Aarhus University, Aarhus, Denmark; 3 Stellar Astrophysics Centre, Department of Physics and Astronomy, Aarhus University, Aarhus, Denmark

**Keywords:** *Bacillus azotoformans*, Denitrification, Codenitrification, Oxygen

## Abstract

*Bacillus azotoformans* MEV2011, isolated from soil, is a microaerotolerant obligate denitrifier, which can also produce N_2_ by co-denitrification. Oxygen is consumed but not growth-supportive. The draft genome has a size of 4.7 Mb and contains key genes for both denitrification and dissimilatory nitrate reduction to ammonium.

## Introduction

Species of the genus *Bacillus* are characterized as Gram-positive, facultative aerobic bacteria capable of forming endospores [1]. In the absence of oxygen, many *Bacillus* species can respire with nitrate instead, employing either dissimilatory nitrate reduction to ammonium or denitrification [2,3]. Despite the widespread occurrence of nitrate-reducing bacilli, their molecular and genetic basis remained poorly investigated [4,5]. Only recently, genome sequencing of two denitrifying type strains, *B. azotoformans* LMG 9581^T^ and *B. bataviensis* LMG 21883^T^, has yielded first insights into the genomic inventory of nitrate reduction and denitrification in Gram-positives [6].

### Classification and features

**Figure 1:**
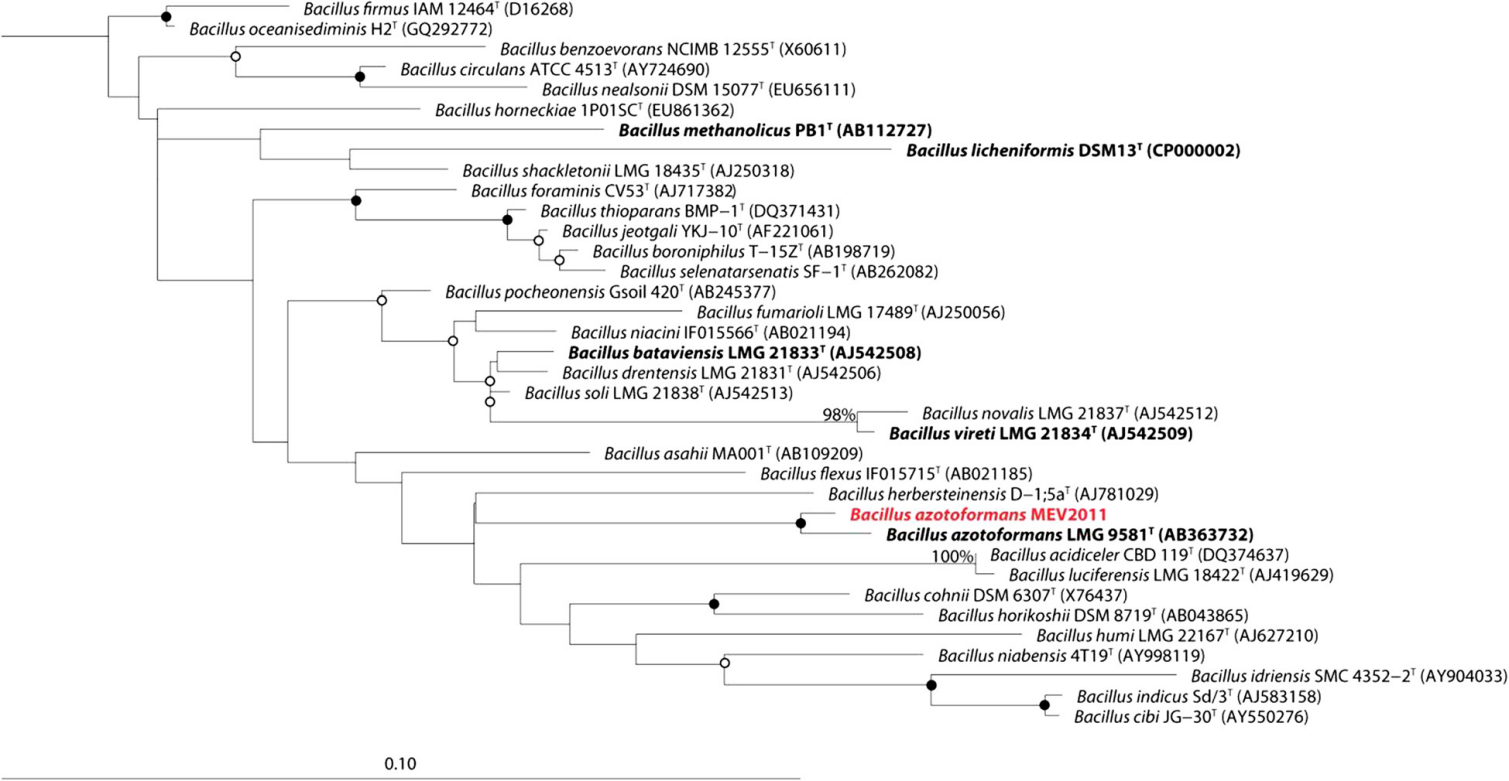
**Phylogenetic tree highlighting the position of** Bacillus azotoformans** MEV2011 (shown in red) relative to closely related (≥95% sequence similarity) type strains within the** Bacillaceae**.** Pre-aligned sequences were retrieved from the Ribosomal Database Project (RDP) [37]. Alignment of the B. azotoformans MEV2011 sequence as well as manual alignment optimization was performed in ARB [38]. The maximum likelihood tree was inferred from 1,478 aligned positions of 16S rRNA gene sequences and calculated based on the General Time Reversible (GTR) model with gamma rate heterogeneity using RAxML 7.4.2 [39]. Type strains with corresponding published genomes are shown in bold face. Open and closed circles indicate nodes with bootstrap support (1,000 replicates ) of 50-80% and >80%, respectively. Escherichia coli ATCC 11577^T^ (X80725) was used to root the tree (not shown). Scale bar, 0.1 substitutions per nucleotide position.

**Figure 2:**
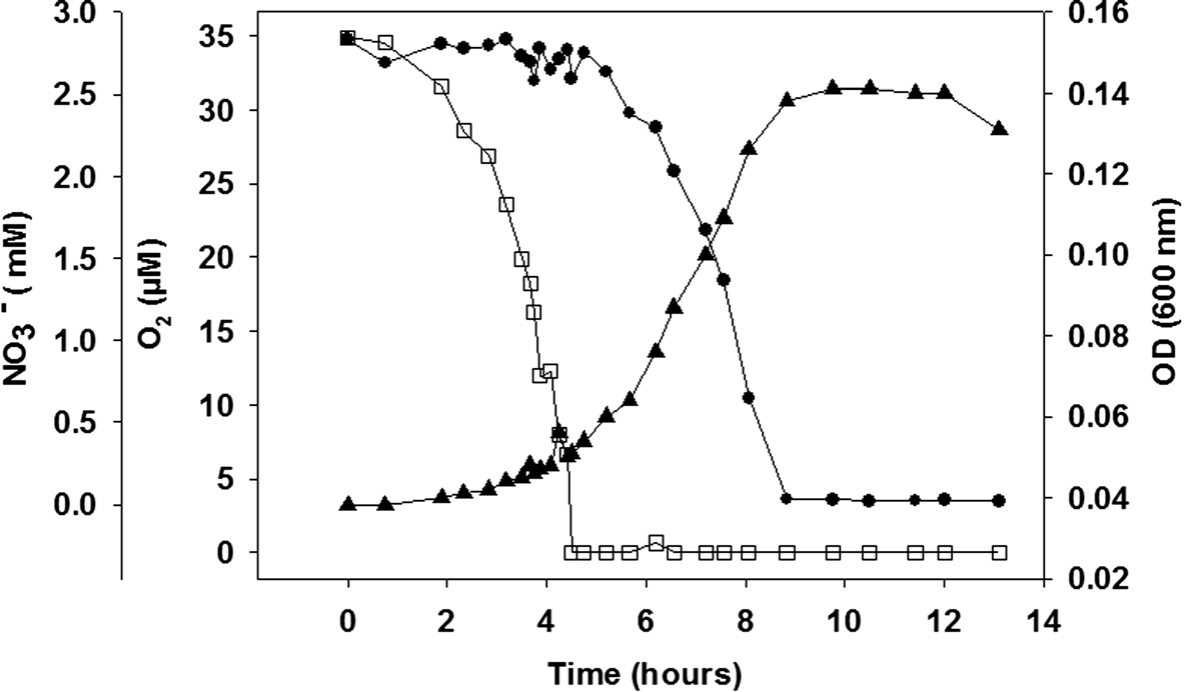
**Consumption of oxygen (□; measured online with an oxygen microsensor) and nitrate (●; measured by HPLC) during growth (▲; OD**_**600**_**) of** B. azotoformans **MEV2011.** No growth was observed at oxygen concentrations >30-35 μM, and the initiation of growth coincided with the first detection of ^30^ N_2_ from ^15^NO_3_^−^ (data not shown), indicating that growth was coupled to denitrification.

**Figure 3:**
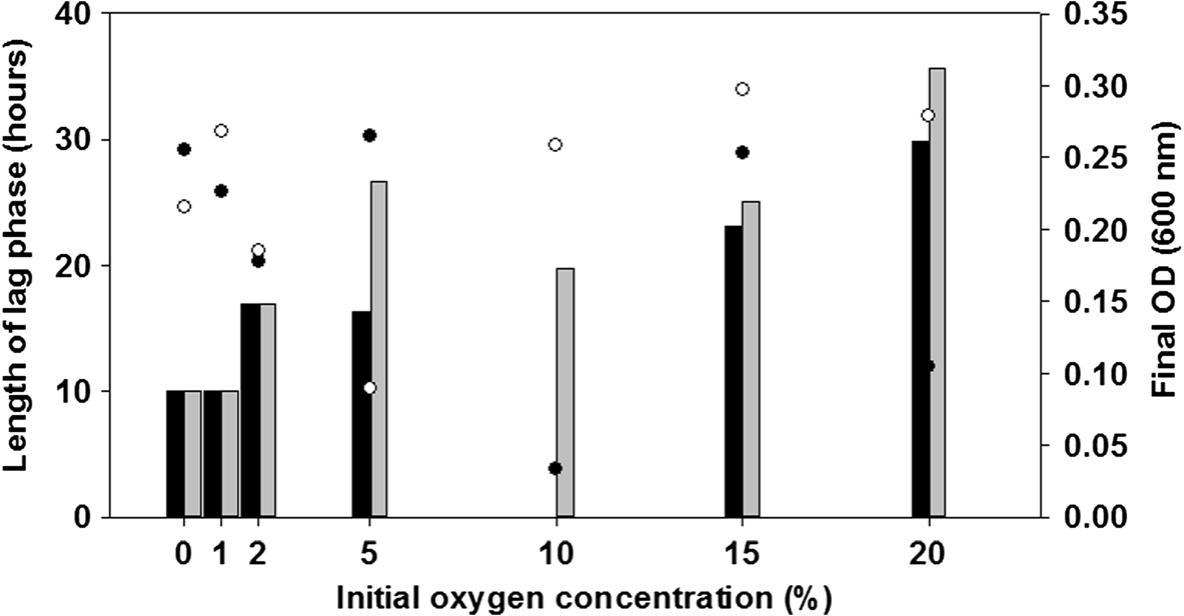
**Length of lag phase (h; bars), and final biomass (OD**_**600; **_**circles) of** B. azotoformans **MEV2011 as function of the initial oxygen concentration in the culture.** Cultures were grown in TSB (10 g L^−1^, Scharlau®) amended with 3 mM KNO_3._ Black and grey bars and circles represent data from replicate incubations. Growth was first detected when oxygen had been consumed to <30–35 μM (see Figure 2), explaining the increasing lag time with increasing oxygen concentrations. The final OD was almost identical in all incubations and unrelated to the initial oxygen concentration, indicating that oxygen did not contribute to biomass production.

*B. azotoformans* MEV2011 (Figure 1) was isolated at 28°C on anoxic King B plates [7] amended with KNO_3_ (5 g L^−1^) from a highly diluted top soil sample at Aarhus University, Denmark. Strain MEV2011 resembles the type strain in its chemoorganotrophic growth on short-chain fatty acids, complete denitrification, and absence of fermentation [8]. However, it differs from the type strain by its inability to grow with oxygen, even though it can tolerate and consume oxygen at atmospheric concentrations. Growth by denitrification (verified by ^15^N incubations; data not shown) starts at microaerobic conditions (<30 μM O_2_; Figure 2), yet the initial presence of oxygen in the growth medium leads to longer lag phases and no increase in final density of the culture (Figure 3); growth without nitrate was never observed. Therefore, we characterize *B. azotoformans* MEV2011 as microaerotolerant obligate denitrifier. In addition, *B. azotoformans* MEV2011 is capable of co-denitrification, a co-metabolic process, in which reduced nitrogen compounds like amino acids or hydroxylamine react with NO^+^ formed during denitrification to produce N_2_O or N_2_[9]; co-denitrification was verified by the mass spectrometric detection of ^30^ N_2_ + ^29^ N_2_ in cultures growing on tryptic soy broth (TSB) and ^15^NO_3_^−^, as suggested in [9]. *B. azotoformans* MEV2011 is available from the BCCM/LMG Bacteria Collection as strain LMG 28302; its general features are summarized in Table 1.

**Table 1:** **Classification and general features of *Bacillus azotoformans *** **MEV2011**[27]

**MIGS ID**	**Property**	**Term**	**Evidence code**^**a**^
	Classification	Domain *Bacteria*	TAS [28]
		Phylum *Firmicutes*	TAS [29]–[31]
		Class *Bacilli*	TAS [32,33]
		Order *Bacillales*	TAS [29,34]
		Family *Bacillaceae*	TAS [29,35]
		Genus *Bacillus*	TAS [29,36]
		Species *Bacillus azotoformans*	TAS [8]
		Strain: MEV2011 (LMG 28302)	IDA
	Gram stain	Variable	IDA
	Cell shape	Rod	IDA
	Motility	Motile	IDA
	Sporulation	Endospore-forming	IDA
	Temperature range	15 – 42°C	IDA
	Optimum temperature	39 – 42°C	IDA
	pH range; Optimum	4–9; 7	IDA
	Carbon source	Malate, acetate, lactate, citrate, succinate, yeast extract	IDA
	Terminal electron acceptor	Nitrate, nitrite, NO, N_2_O (O_2_ is reduced but does not support growth)	IDA
MIGS-6	Habitat	Soil	IDA
MIGS-6.3	Salinity	0–3% NaCl (w/v)	IDA
MIGS-22	Oxygen requirement	Anaerobic, microaerotolerant	IDA
MIGS-15	Biotic relationship	Free-living	IDA
MIGS-14	Pathogenicity	Non-pathogen	IDA
MIGS-4	Geographic location	Denmark/Aarhus University campus, Aarhus	IDA
MIGS-5	Sample collection	2011-02-01	IDA
MIGS-4.1	Latitude	56° 10’ 0.12” N	IDA
MIGS-4.2	Longitude	10° 12’ 6.12” E	IDA
MIGS-4.4	Altitude	38.6 m	IDA

^a^Evidence codes - IDA: Inferred from Direct Assay; TAS: Traceable Author Statement (i.e., a direct report exists in the literature); NAS: Non-traceable Author Statement (i.e., not directly observed for the living, isolated sample, but based on a generally accepted property for the species, or anecdotal evidence). These evidence codes are from the Gene Ontology project [10].

### Genome sequencing and annotation

#### Genome project history

*Bacillus azotoformans* MEV2011 was selected for whole genome sequencing based on its unusual “obligate” denitrifying phenotype, i.e. its inability to grow under oxic conditions, together with its co-denitrifying capacity. Comparing the genome of strain MEV2011 to that of the oxygen-respiring and conventionally denitrifying type strain [8] may provide insights into the molecular basis of its metabolic features. The draft genome sequence was completed on July 20, 2013. The genome project is deposited in the Genomes OnLine Database (GOLD) as project Gp0043190. Raw sequencing reads have been deposited at the NCBI Sequence Read Archive (SRA) under the experiment numbers SRX527325 (100 bp library) and SRX527326 (400 bp library). This Whole Genome Shotgun project has been deposited at GenBank under the accession number JJRY00000000. The version described in this paper is version 1. Table 2 presents the project information and its association with MIGS version 2.0 compliance [27].

**Table 2:** Project information

**MIGS ID**	**Property**	**Term**
MIGS-31	Finishing quality	High quality draft
MIGS-28	Libraries used	IonTorrent 100 bp and 400 bp single end reads
MIGS-29	Sequencing platforms	IonTorrent PGM
MIGS-31.2	Fold coverage	110×
MIGS-30	Assemblers	Newbler 2.6, MIRA 3.9.18, Sequencher 5.0.1
MIGS-32	Gene calling method	Prodigal
	Locus Tag	M670
	Genbank ID	JJRY00000000
	Genbank Date of Release	2014-06-16
	GOLD ID	Gi0050495
	BIOPROJECT	PRJNA209301
	Project relevance	Environmental, co-denitrification
MIGS 13	Source Material Identifier	LMG 28302

### Growth conditions and genomic DNA preparation

*B. azotoformans* MEV2011 was grown at 28°C in N_2_-flushed TSB (10 g L^−1^, Scharlau®) amended with KNO_3_ (3 g L^−1^). DNA was extracted using the DNeasy Blood & Tissue kit (Qiagen®).

### Genome sequencing and assembly

Sequencing of the *B. azotoformans* MEV2011 genome was performed with an Ion Torrent PGM sequencer (Life Sciences) using 100 and 400 bp sequencing chemistries. Sequencing libraries were prepared using Ion Xpress™ Plus Fragment Library Kits (Life Sciences), and Ion OneTouch™ Template Kits (Life Sciences). Sequencing of the 100 bp library generated 442,853 reads (representing 42 Mbp of sequence information), while sequencing of the 400 bp library generated 2,401,947 reads (477 Mbp). Together, both libraries achieved a genome coverage of *c.* 110× for an estimated genome size of 4.7 Mbp. The reads were quality trimmed using the prinseq-lite.pl script [11] with the following parameters; reads generated with 100 bp chemistry: -*min_len 50 -trim_to_len 110 -trim_left 15 -trim_qual_right 20 -trim_qual_window 4 -trim_qual_type mean*; reads generated with 400 bp chemistry: -*min_len 50 -trim_to_len 400 -trim_left 15 -trim_qual_right 20 -trim_qual_window 4 -trim_qual_type mean*. The trimmed reads (2,491,456 reads representing 444 Mbp) were assembled using MIRA 3.9.18 [12] with the following parameters: *job = genome,denovo,accurate*; *technology = iontor.* In parallel, the reads were also assembled using *Newbler* 2.6 (Roche) with the following parameters: *-mi 96 –ml 50* (i.e. 96% minimum sequence similarity and 50 bp minimum overlap). Contigs shorter than 1,000 bp were removed from both assemblies. All remaining contigs were trimmed by 50 bp from the 5’ and the 3’ ends using the prinseq-lite.pl script in order to remove error-prone contig ends. The two assemblies were merged and manually inspected using Sequencher 5.0.1 (Genecodes). In cases where the bases of the two assemblies disagreed, the *Newbler* variant was preferred. Contigs not contained in both assemblies were removed from the data set. The final assembly yielded 56 contigs representing 4.7 Mbp of sequence information.

### Genome annotation

The draft genome was auto-annotated using the standard operation procedure of the Integrated Microbial Genomes Expert Review (IMG-ER) platform developed by the Joint Genome Institute, Walnut Creek, CA, USA [13]. In short, CRISPR regions were identified by CRT [14] and PILERCR [15], tRNAs were identified by tRNAScan-SE-1.23 [16], rRNAs were identified by RNAmmer [17], and finally all other genes were identified by Prodigal [18]. Functional annotation was based on gene comparisons with the KEGG database (release 63.0, July 1, 2012) [19], the PFAM database (version 25.0, March 30, 2011) [20], the cluster of orthologous groups (COG) [21] database, and the TIGRfam database (release 11.0, August 3, 2011) [22].

### Genome properties

The MEV2011 draft genome is 4,703,886 bp long and comprises 56 contigs ranging in size from 1,773 to 525,568 bp, with an overall GC content of 37.49% (Table 3). Of the 4,986 predicted genes, 4,809 (96.45%) are protein-coding genes, and 177 are RNAs. Of the RNAs, 94 are tRNAs, and 37 are rRNAs. The number of 5S rRNAs as well as the number of partial 16S and 23S rRNA genes indicates a total of 11 rRNA operons. Most (75.3%) protein-coding genes were assigned to putative functions. The distribution of genes into COG functional categories is presented in Table 4.

**Table 3:** Nucleotide content and gene count levels of the genome

**Attribute**	**Value**	**% of total**^**a**^
Genome size (bp)	4,703,886	100
DNA coding (bp)	4,075,859	86.7
DNA G + C (bp)	1,763,498	37.5
DNA scaffolds	56	100
Total genes	4,986	100
Protein coding genes	4,809	96.5
RNA genes	177	3.6
Pseudo genes	0	0
Genes in internal clusters	3,448	69.1
Genes with function prediction	3,755	75.3
Genes assigned to COGs	2,809	56.3
Genes with Pfam domains	3,890	78.0
Genes with signal peptides	182	3.7
Genes with transmembrane helices	1,233	24.7
CRISPR repeats	4	-

a)The total is based on either the size of the genome in base pairs or the total number of protein coding genes in the annotated genome.

**Table 4:** Number of genes associated with general COG functional categories

**Code**	**Value**	**% age**^**a**^	**Description**
A	0	0	RNA processing and modification
J	160	3.32	Translation, ribosomal structure and biogenesis
K	246	5.11	Transcription
L	185	3.85	Replication, recombination and repair
B	1	0.02	Chromatin structure and dynamics
D	34	0.70	Cell cycle control, Cell division, chromosome partitioning
V	45	0.94	Defense mechanisms
T	207	4.30	Signal transduction mechanisms
M	123	2.56	Cell wall/membrane biogenesis
N	77	1.60	Cell motility
U	50	1.04	Intracellular trafficking and secretion
O	106	2.20	Posttranslational modification, protein turnover, chaperones
C	216	4.49	Energy production and conversion
G	136	2.83	Carbohydrate transport and metabolism
E	299	6.22	Amino acid transport and metabolism
F	67	1.39	Nucleotide transport and metabolism
H	144	2.99	Coenzyme transport and metabolism
I	135	2.81	Lipid transport and metabolism
P	173	3.60	Inorganic ion transport and metabolism
Q	81	1.68	Secondary metabolites biosynthesis, transport and catabolism
R	350	7.28	General function prediction only
S	291	6.05	Function unknown
-	2,177	45.27	Not in COGs

a)The total is based on the total number of protein coding genes in the annotated genome.

## Insights from the genome sequence

Overall, the genome of the novel strain MEV2011 appeared highly similar to that of the *B. azotoformans* type strain LMG 9581^T^[8]. *In silico* DNA–DNA hybridization (DDH) was performed for the assembled MEV2011 genome against the published genome of LMG 9581^T^ (Acc. number NZ_AJLR00000000); the contigs of *B. azotoformans* LMG 9581^T^ were assembled into one FASTA file before uploading to the online genome-to-genome calculator provided by the DSMZ [23]. Using the GGDC 2.0 model, DHH estimates were always >70%, irrespective of the formula used for computing DHH, and with probabilities between 78 and 87%. These results confirm that MEV2011 is a novel strain of the species *B. azotoformans*.

Just as *B. azotoformans* LMG 9581^T^, strain MEV2011 carries multiple copies of key denitrification genes, encodes both membrane-bound and periplasmic nitrate reductases, and the key genes for nitrite reduction to both NO (in denitrification) and ammonium (in DNRA); see (Additional file 1: Table S1) and reference [6] for details. Modularity and redundancy in nitrate reduction pathways has also been observed in other *Bacillus* species (e.g. *B. bataviensis*[6], *Bacillus* sp. strain ZYK [24], *Bacillus* sp. strain 1NLA3E [25]), and may be a general feature of nitrate-reducing members of this genus.

All genes essential for aerobic respiration were identified, including those for terminal oxidases (see Additional file 1: Table S2) and for detoxifying reactive oxygen species (see Additional file 1: Table S3). Therefore, the inability of *B. azotoformans* MEV2011 to grow with oxygen remains a conundrum and in some way resembles that of various sulfate-reducing bacteria, which also consume oxygen and even produce ATP during oxic respiration but are unable to grow in the presence of oxygen [26].

## Conclusion

Based on our whole genome comparison, the microaerotolerant obligate (co-) denitrifying *Bacillus* sp. MEV2011 (LMG 28302) is a novel strain of *Bacillus azotoformans*, with similar redundancy in its nitrate reduction pathways, including the potential for DNRA, and a complete set of genes for oxic respiration and oxygen detoxification; its inability to grow with oxygen remains enigmatic.

## Abbreviations

DNRA: Dissimilatory nitrate reduction to ammonium.

## Competing interests

The authors declare that they have no competing interests.

## Authors’ contributions

KF and AS designed research, MN isolated and characterized strain MEV2011 and carried out the genome sequencing, LS performed bioinformatics analyses, all authors analyzed data, MN and LS wrote the manuscript with help of AS and KF, all authors read and approved the final manuscript.

## Supplementary Material

Additional file 1: Table S1 Overview of the genomic inventory for dissimilatory nitrogen transformations in Bacillus azotoformans MEV2011. **Table S2.** Overview of the genomic inventory for enzymatic reduction of O_2_ and ATP synthase in Bacillus azotoformans MEV2011. **Table S3.** Overview of the genomic inventory for the detoxification of reactive oxygen species in Bacillus azotoformans MEV2011. Click here for file
