# Supplementary material for: Draft genome sequence of Bacillus azotoformans MEV2011, a (Co-) denitrifying strain unable to grow with oxygen
Source: Stand Genomic Sci. 2015 Jan 21;9:23. doi: 10.1186/1944-3277-9-23 (PMC4322954; doi:10.1186/1944-3277-9-23)
Supplement: Additional file 1: Table S1 — Overview of the genomic inventory for dissimilatory nitrogen transformations in Bacillus azotoformans MEV2011. Table S2. Overview of the genomic inventory for enzymatic reduction of O2 and ATP synthase in Bacillus azotoformans MEV2011. Table S3. Overview of the genomic inventory for the detoxification of reactive oxygen species in Bacillus azotoformans MEV2011. [file 1944-3277-9-23-S1.pdf]

Maja Nielsen, Lars Schreiber, Kai Finster, and Andreas Schramm:

Draft Genome Sequence of *Bacillus azotoformans* MEV2011, a (Co-) Denitrifying Strain Unable to Grow with Oxygen

Supplementary Tables

Table S1. Overview of the genomic inventory for dissimilatory nitrogen transformations in *Bacillus azotoformans* MEV2011 (according to the IMG autoannotation).

Function	Gene	Locus Tag	Gene Coordinates	GenBank Identifier (pBLAST best hit)	% ID
Cytoplasmic dissimilatory nitrate reduction	*narG1*	M670_00379	330131..333814(3684bp)	WP_003331056.1 *Bacillus azotoformans* LMG9581	99
	narH1	M670_00378	328591..330141(1551bp)	WP_003331055.1 *Bacillus azotoformans* LMG9581	99
	narJ1	M670_00377	328056..328604(549bp)	WP_003331054.1 *Bacillus azotoformans* LMG9581	99
	narI1	M670_00376	327296..328012 (717bp)	WP_003331053.1 *Bacillus azotoformans* LMG9581	97
	narJ2	M670_03226	120251..120832 (582bp)	YP_001124780.1 *Geobacillus thermodenitrificans* NG80-2	36
	narG2	M670_03224	15014..118781 (3768bp)	YP_516567.1 *Desulfitobacterium hafniense* Y51	70
	narH2	M670_03225	118771..120288 (1518bp)	YP_003242070.1 *Paenibacillus sp.* Y412MC10	70
	narI2	M670_03227	120835..121515 (681bp)	YP_252572.1 *Staphylococcus haemolyticus* JCSC1435	56
	narJ3	M670_04859	10048..10602 (555bp)	WP_003331449.1 *Bacillus azotoformans* LMG9581	98
	narH3	M670_04860	10048..10602 (1467bp)	WP_003331450.1 *Bacillus azotoformans* LMG9581	98
	narI3	M670_04858	9362..10051 (690bp)	WP_003331448.1 *Bacillus azotoformans* LMG9581	100
	narG3	M670_04861	2138..15821(3684bp)	WP_003331451.1 *Bacillus azotoformans* LMG9581	98

Function	Gene	Locus Tag	Gene Coordinates	GenBank Identifier (pBLAST best hit)	% ID
Periplasmic dissimilatory nitrate reduction	*napB*	M670_01957	102803..103222 (420bp)	WP_003332435.1 *Bacillus azotoformans* LMG9581	99
	napG1	M670_01955	99581..100177 (597bp)	WP_003332433.1 *Bacillus azotoformans* LMG9581	99
	napA	M670_01956	100253..102790 (2538bp)	WP_003332434.1 *Bacillus azotoformans* LMG9581	100
	napD	M670_01958	103267..103524 (258bp)	WP_003332436.1 *Bacillus azotoformans* LMG9581	97
	napG2	M670_01959	103530..104099 (570bp)	WP_003332437.1 *Bacillus azotoformans* LMG9581	99
	napH	M670_02517	30733..31632 (900bp)	WP_003332276.1 *Bacillus azotoformans* LMG9581	99
	moa	M670_01954	98302..99333 (1032bp)	WP_003332432.1 *Bacillus azotoformans* LMG9581	99

Function	Gene	Locus Tag	Gene Coordinates	GenBank Identifier (pBLAST best hit)	% ID
Dissimilatory reduction to NO	*nirK*	M670_01705	69499..70560 (1062bp)	WP_003329883.1 *Bacillus azotoformans* LMG9581	99

Function	Gene	Locus Tag	Gene Coordinates	GenBank Identifier (pBLAST best hit)	% ID
Dissmilatory nitrite reduction to ammonium	*nrfH*	M670_01133	104203..104718 (516bp)	WP_003329814.1 *Bacillus azotoformans* LMG9581	100
	nrfA	M670_01132	102744..104201 (1458bp)	WP_003329815.1 *Bacillus azotoformans* LMG9581	100

Function	Gene	Locus Tag	Gene Coordinates	GenBank identifier (pBLAST best hit)	% ID
Dissimilatory quinol-dependent nitric oxide reduction	*qnorB1*	M670_01459	65358..67619 (2262bp)	WP_003329195.1 *Bacillus azotoformans* LMG9581	99
	qnorB2	M670_00384	335562..337925 (2364bp)	WP_003331062.1 *Bacillus azotoformans* LMG9581	99

Dissimilatory menaquinol/cytc-dependent nitric oxide reduction (Type I)	*cbaB1*	M670_03526	55005..55523 (519bp)	WP_003330136.1 *Bacillus azotoformans* LMG9581	99
	cbaA1	M670_03525	53497..54978 (1482bp)	WP_003330135.1 *Bacillus azotoformans* LMG9581	99
	senC1	M670_03524	52899..53504 (606bp)	WP_003330134.1 *Bacillus azotoformans* LMG9581	99
	cbaD1	M670_03523	52309..52872 (564bp)	WP_003330133.1 *Bacillus azotoformans* LMG9581	88
	cbaC2	M670_04975	4839..5411 (573bp)	WP_003330503.1 *Bacillus azotoformans* LMG9581	98
Dissimilatory menaquinol/cytc-dependent nitric oxide reduction (Type II)	*senC2*	M670_04974	4204..4839 (636bp)	WP_003330502.1 *Bacillus azotoformans* LMG9581	98
	cbaB2	M670_04972	1938..2399 (462bp)	WP_003330500.1 *Bacillus azotoformans* LMG9581	96
	cbaA2	M670_04973	2414..4117 (1704bp)	WP_003330501.1 *Bacillus azotoformans* LMG9581	94

**Function**	**Gene**	**Locus Tag**	**Gene Coordinates**	**GenBank Identifier (pBLAST best hit)**	% ID
	nosF	M670_01450	54548..55465 (918bp)	WP_003329185.1 *Bacillus azotoformans* LMG9581	98
Dissimilatory nitrous oxide reduction	*nosY*	M670_01449	53709..54551 (843bp)	WP_003329184.1 *Bacillus azotoformans* LMG9581	99
	nosL	M670_01448	53225..53734 (510bp)	WP_003329183.1 *Bacillus azotoformans* LMG9581	96
	nosD	M670_01447	51885..53228 (1344bp)	WP_003329182.1 *Bacillus azotoformans* LMG9581	96
	nosZ	M670_01444	49300..51150 (1851bp)	WP_003329179.1 *Bacillus azotoformans* LMG9581	99
	nosC	M670_01443	48814..49275 (462bp)	WP_003329178.1 *Bacillus azotoformans* LMG9581	94
	nosC	M670_03087	131955..132401 (447bp)	WP_003330275.1 *Bacillus azotoformans* LMG9581	95

	nosZ	M670_03086	130050..131930 (1881bp)	WP_003330276.1 *Bacillus azotoformans* LMG9581	99
	nosD	M670_03084	128108..129433 (1326bp)	WP_003330278.1 *Bacillus azotoformans* LMG9581	99
	nosD	M670_00060	54903..56246 (1344bp)	WP_003332806.1 *Bacillus azotoformans* LMG9581	100
	nosL	M670_00059	54421..54906 (486bp)	WP_003332807.1 *Bacillus azotoformans* LMG9581	99
	nosY	M670_00058	53550..54380 (831bp)	WP_003332808. *Bacillus azotoformans* LMG9581	97
	nosF	M670_00057	52814..53560 (747bp)	WP_003332809.1 *Bacillus azotoformans* LMG9581	97
	nosL	M670_00056	51729..52448 (720bp)	WP_003332811.1 *Bacillus azotoformans* LMG9581	97
	nosC	M670_00026	24574..25008 (435bp)	WP_003332840.1 *Bacillus azotoformans* LMG9581	99
	nosZ	M670_00025	22681..24558 (1878bp)	WP_003332841.1 *Bacillus azotoformans* LMG9581	100
	nosD	M670_00023	20637..22052 (1416bp)	WP_003332843.1 *Bacillus azotoformans* LMG9581	99
	nosY	M670_00022	19840..20637 (798bp)	WP_003332844.1 *Bacillus azotoformans* LMG9581	100
	nosF	M670_00021	19172..19843 (672bp)	WP_003332845.1 *Bacillus azotoformans* LMG9581	100

**Function**	**Gene**	**Locus Tag**	**Gene Coordinates**	**GenBank identifier (pBLAST best hit)**	**% ID**
Nitrate/Nitrite transporter	*narK*	M670_03223	113794..115014 (1221bp)	WP_019240754.1 *Bacillus massilioanorexius*	75
	nirC	M670_04679	22520..23290 (771bp)	WP_003331452 *Bacillus azotoformans* LMG9581	97

Table S2. Overview of the genomic inventory for enzymatic reduction of O_2 and ATP synthase in *Bacillus azotoformans* MEV2011 (according to the IMG autoannotation). Genes encoding complex I-IV in the electron transport chain and an ATPase were identified. In addition, genes were found encoding an alternative terminal oxidase.

**Locus Tag**	**Gene Product Name**	**Gene Coordinates**	**pBLAST best hit**	
	**Complex I**		**GenBank Identifier**	**%ID**
M670_02616	NADH dehydrogenase subunit A [EC: 1.6.5.3]	119047...119421 (375bp)	WP_003333358.1 *Bacillus azotoformans* LMG9581	100
M670_02617	NADH dehydrogenase subunit B [EC: 1.6.5.3]	119412…119933 (522bp)	WP_003333357.1 *Bacillus azotoformans* LMG9581	99
M670_02618	NADH dehydrogenase subunit C [EC: 1.6.5.3]	119961…121145 (1185bp)	WP_003333355.1 *Bacillus azotoformans* LMG9581	97
M670_02619	NADH dehydrogenase subunit D [EC: 1.6.5.3]	121149…122252 (1104bp)	WP_003333354.1 *Bacillus azotoformans* LMG9581	100
M670_02620	NADH dehydrogenase subunit H [EC: 1.6.5.3]	122252…123256 (1005bp)	WP_017756590.1 *Bacillus* sp. ZYK	83
M670_02621	NADH dehydrogenase subunit I [EC: 1.6.5.3]	123299…123718 (420bp)	WP_003333353.1 *Bacillus azotoformans* LMG9581	100
M670_02622	NADH dehydrogenase subunit J [EC: 1.6.5.3]	123715…124230 (516bp)	WP_003333352.1 *Bacillus azotoformans* LMG9581	96
M670_02623	NADH dehydrogenase subunit K [EC: 1.6.5.3]	124223…124537 (315bp)	WP_003333351.1 *Bacillus azotoformans* LMG9581	100
M670_02624	NADH dehydrogenase subunit L [EC: 1.6.5.3]	124556…126454 (1899bp)	WP_003333350.1 *Bacillus azotoformans* LMG9581	99
M670_02625	NADH dehydrogenase subunit M [EC: 1.6.5.3]	126451…127959 (1509bp)	WP_003333349.1 *Bacillus azotoformans* LMG9581	99
M670_02626	NADH dehydrogenase subunit N [EC: 1.6.5.3]	127966…19486 (1521bp)	WP_003333348.1 *Bacillus azotoformans* LMG9581	99
M670_00090	NADH dehydrogenase, FAD-containing subunit [EC: 1.6.99.3]	77922…79145 (1224bp)	WP_003332777.1 *Bacillus azotoformans* LMG9581	99

**Locus Tag**	**Gene Product Name**	**Gene Coordinates**	**pBLAST best hit**	
	**Complex II**		**GenBank Identifier**	**%ID**
M670_01296	succinate dehydrogenase subunit B [EC: 1.3.99.1]	268315…269070 (756bp)	WP_003331006.1 *Bacillus azotoformans* LMG9581	99
M670_01297	succinate dehydrogenase subunit A [EC: 1.3.99.1]	269073…270863 (1791bp)	WP_003331007.1 *Bacillus azotoformans* LMG9581	98
M670_01298	succinate dehydrogenase subunit C [EC: 1.3.5.1]	271108..271737 (630bp)	WP_003331008.1 *Bacillus azotoformans* LMG9581	100

Locus Tag	Gene Product Name	Gene Coordinates	pBLAST best hit	
	**Complex III**		**GenBank Identifier**	**%ID**
M670_00552	menaquinol-cytochrome c reductase cytochrome b/c subunit	458363...459136 (774bp)	WP_003331133.1 *Bacillus azotoformans* LMG9581	100
M670_00553	menaquinol-cytochrome c reductase cytochrome b subunit	459168...459842 (675bp)	WP_003331134.1 *Bacillus azotoformans* LMG9581	100
M670_00554	Rieske Fe-S protein [EC: 1.10.2.-]	459857...460354 (498bp)	WP_003331135.1 *Bacillus azotoformans* LMG9581	99

Locus Tag	Gene Product Name	Gene Coordinates	pBLAST best hit	
	**Complex IV (cytochrome c oxidase)**		**GenBank Identifier**	**%ID**
M670_04800	cytochrome c oxidase assembly factor CtaG	3158...4060 (903bp)	WP_003329986.1 *Bacillus azotoformans* LMG9581	95
M670_04801	cytochrome c oxidase, subunit IVB [EC: 1.9.3.1]	4101…4433 (333bp)	WP_003329987.1 *Bacillus azotoformans* LMG9581	99
M670_04802	Heme/copper-type cytochrome/quinol oxidase, subunit 3 [EC: 1.9.3.1]	4439…5059 (621bp)	WP_003329988.1 *Bacillus azotoformans* LMG9581	100
M670_04803	Heme/copper-type cytochrome/quinol oxidases, subunit 1 [EC: 1.9.3.1]	5059…6930 (1872bp)	WP_003329990.1 *Bacillus azotoformans* LMG9581	99
M670_04804	cytochrome c oxidase, subunit II [EC: 1.9.3.1]	6947…7990 (1044bp)	WP_003329991.1 *Bacillus azotoformans* LMG9581	99
M670_04805	cyoE protoheme IX farnesyltransferase [EC:2.5.1.-]	8424...9167(744bp)	WP_003329992.1 *Bacillus azotoformans* LMG9581	99

Locus Tag	Gene Product Name	Gene Coordinates	pBLAST best hit	
	**Complex IV (alternative terminal oxidase)**		**GenBank Identifier**	**%ID**
M670_01069	cytochrome aa3-600 menaquinol oxidase subunit IV [EC:1.10.3.12]	49816..50109 (294bp)	WP_003331328.1 *Bacillus azotoformans* LMG9581	98
M670_01068	cytochrome aa3-600 menaquinol oxidase subunit III [EC:1.10.3.12]	49217...49813 (597bp)	WP_003331327.1 *Bacillus azotoformans* LMG9581	100
M670_01067	cytochrome aa3-600 menaquinol oxidase subunit I [EC:1.10.3.12]	47268...49217 (1950bp)	WP_003331326.1 *Bacillus azotoformans* LMG9581	99
M670_01066	cytochrome aa3-600 menaquinol oxidase subunit II [EC:1.10.3.12]	46351...47265 (915bp)	WP_003331325.1 *Bacillus azotoformans* LMG9581	98

Locus Tag	Gene Product Name	Gene Coordinates	pBLAST best hit	
	**ATPase**		**GenBank Identifier**	**%ID**
M670_04237	ppk polyphosphate kinase [EC:2.7.4.1]	21328…23442 (2115bp)	WP_003330039.1 *Bacillus azotoformans* LMG9581	99
M670_00299	pyrophosphatase PpaX [EC:3.6.1.1]	247358…248005 (648bp)	WP_003330763.1 *Bacillus azotoformans* LMG9581	99
M670_02607	atpB F-type H+-transporting ATPase subunit a [EC:3.6.3.14]	111451…112161 (711bp)	WP_003333362.1 *Bacillus azotoformans* LMG9581	99
M670_02608	ATP synthase F0 subcomplex C subunit [EC:3.6.3.14]	112216…112434 (219bp)	WP_003333365.1 *Bacillus azotoformans* LMG9581	100
M670_02609	ATP synthase F0 subcomplex B subunit [EC:3.6.3.14]	112693…113223 (531bp)	WP_003333364.1 *Bacillus azotoformans* LMG9581	100
M670_02610	ATP synthase F1 subcomplex delta subunit [EC:3.6.3.14]	113220…113765 (546bp)	WP_003333363.1 *Bacillus azotoformans* LMG9581	100
M670_02611	ATP synthase F1 subcomplex alpha subunit [EC:3.6.3.14]	113783…115291 (1509bp)	WP_003333362.1 *Bacillus azotoformans* LMG9581	100
M670_02612	ATP synthase F1 subcomplex gamma subunit [EC:3.6.3.14]	115374…115234 (861bp)	WP_003333361.1 *Bacillus azotoformans* LMG9581	99
M670_02613	ATP synthase F1 subcomplex beta subunit [EC:3.6.3.14]	116337…117749 (1413bp)	WP_003333360.1 *Bacillus azotoformans* LMG9581	99
M670_02615	ATP synthase F1 subcomplex epsilon subunit [EC:3.6.3.14]	117977…118378 (402bp)	WP_003333359.1 *Bacillus azotoformans* LMG9581	99
M670_00902	type III secretion system ATPase, FliI/YscN [EC: 3.6.3.15]	272821…274131 (1311bp)	WP_003332537.1 *Bacillus azotoformans* LMG9581	99

Table S3. Overview of the genomic inventory for the detoxification of reactive oxygen species in *Bacillus azotoformans* MEV2011 (according to the IMG autoannotation).

Locus Tag	Gene Product Name	Gene Coordinates	pBLAST best hit	
			**GenBank Identifier**	**%ID**
M670_04927	SOD2 superoxide dismutase, Fe-Mn family [EC:1.15.1.1]	11018…11629 (612bp)	WP_003332388.1 *Bacillus azotoformans*	100
M670_01567	Cu2+/Zn2+ superoxide dismutase SOD1	156379…157017 (639bp)	WP_003331991.1 *Bacillus azotoformans*	99
M670_01373	tpx thiol peroxidase, atypical 2-Cys peroxiredoxin [EC:1.11.1.15]	346493…34993 (501bp)	WP_003347404.1 *Bacillus methanolicus*	86
M670_04941	glutathione peroxidase [EC:1.11.1.9]	6177…6740 (564bp)	WP_003331960.1 *Bacillus azotoformans*	97
M670_00605	Mn-containing catalase	503460..504029 (570bp)	WP_017755616.1 *Bacillus* sp. ZYK	91
